# Graphene Oxide-Gallic Acid Nanodelivery System for Cancer Therapy

**DOI:** 10.1186/s11671-016-1712-2

**Published:** 2016-11-08

**Authors:** Dena Dorniani, Bullo Saifullah, Farahnaz Barahuie, Palanisamy Arulselvan, Mohd Zobir Bin Hussein, Sharida Fakurazi, Lance J. Twyman

**Affiliations:** 1 Department of Chemistry, University of Sheffield, Dainton Building, Brook Hill, Sheffield, S3 7HF UK; 2 Materials Synthesis and Characterization Laboratory, Institute of Advanced Technology, University Putra Malaysia, 43400 Serdang, Malaysia; 3 Department of Chemistry, Zabol University of Medical Sciences, Zabol, Iran; 4 Laboratory of Vaccines and Immunotherapeutics, Institute of Bioscience, University Putra Malaysia, 43400 Serdang, Malaysia; 5 Department of Human Anatomy, Faculty of Medicine and Health Sciences, University Putra Malaysia, 43400 Serdang, Malaysia

**Keywords:** Drug delivery systems, Graphene oxide, Gallic acid, Controlled-release, Cytotoxicity

## Abstract

Despite the technological advancement in the biomedical science, cancer remains a life-threatening disease. In this study, we designed an anticancer nanodelivery system using graphene oxide (GO) as nanocarrier for an active anticancer agent gallic acid (GA). The successful formation nanocomposite (GOGA) was characterized using XRD, FTIR, HRTEM, Raman, and UV/Vis spectroscopy. The release study shows that the release of GA from the designed anticancer nanocomposite (GOGA) occurs in a sustained manner in phosphate-buffered saline (PBS) solution at pH 7.4. In in vitro biological studies, normal fibroblast (3T3) and liver cancer cells (HepG2) were treated with different concentrations of GO, GOGA, and GA for 72 h. The GOGA nanocomposite showed the inhibitory effect to cancer cell growth without affecting normal cell growth. The results of this research are highly encouraging to go further for in vivo studies.

## Background

Despite concerted research effort to fight various diseases to improve human health, cancer still remains as one of the biggest challenges for human beings [1, 2]. Two major methods can be used to overcome this problem. The first is to develop and synthesize new anticancer drugs. And the second is to develop new and effective delivery systems with the ability to improve the therapeutic profile and efficacy of existing therapeutic agents [3, 4]. Therefore, various nanocarrier-based therapeutic and diagnostic agents such as liposome, carbon nanotubes, polymeric nanoparticles, and dendrimers have been extensively studied to prolong the half-life of drug systemic circulation and lower frequency of administration to minimize systemic side effects of drugs. Drugs can be loaded or attached via different mechanisms, such as embedding, physical absorption, and hydrogen-bonding interactions [5] resulting in the sustained release of drugs over longer periods of time [2, 6, 7].

Recently, graphene and its chemically oxidized derivative, graphene oxide (GO) (a 2D hydrophilic carbon material) [8–11], have been investigated for targeted drug delivery for cancer therapy [12–19], biosensing, and cancer photothermal therapy, as they have a proportionally large surface area than many other materials, ample functional groups on the surface, good photothermal properties, and/or low cytotoxicity [20, 21]. The amphiphilic and planar structure of GO enable it to incorporate hydrophilic and/or hydrophobic biomolecules which gives the opportunity to prevent its instability in a medium [15, 18, 22]. In addition, the large surface area and oxygen-containing functional groups of GO such as phenol hydroxyl, epoxy, and carboxylic groups make it ideal for providing high drug loading efficiency, good dispersion, and easily functionalization [2, 23].

Gallic acid (GA), a polyhydroxylphenolic compound, which has a wide range of biological applications such as antiviral, antibacterial, antimelanogenic, antimutagenic, anti-inflammatory, and anticancer activity, in a range of cells is distributed in a variety of fruits, plants, and foods [24–29]. In this study, we have selected GA as a model drug to be loaded onto the as-prepared GO nanocarrier to form a new nanocomposite (GOGA) for active drug delivery and specific cell targeting system in normal fibroblasts (3T3) and in liver cancer cells, HepG2. The results from the X-ray diffraction (XRD), Fourier transform infrared (FTIR), Raman spectroscopy, TGA/DTG, anticancer evaluation, and cytotoxicity as well as release property of GA from GOGA nanocomposite into aqueous media will be also discussed.

## Methods

### Materials

Graphite flakes with 100 meshes (Sigma), potassium permanganate (99 %), sulphuric acid (95–97 %), hydrochloric acid (37 %), diethyl ether, ortho-phosphoric acid (85 %) and hydrogen peroxide (35 %) by Riendemann chmidt, gallic acid (97 % purity) (Sigma-Aldrich), and ethyl alcohol (99.7 % *v*/*v*) (Hayman) were used in this work. All the aqueous solutions were prepared using deionized water (18.2 M Ω cm^−1^).

### Characterization

The synthesized products were characterized by powder X-ray diffraction (XRD-6000 diffractometer, Shimadzu, Tokyo, Japan), with CuK_α_ radiation (*λ* = 1.5406 Å) at 40 kV and 30 mA. FTIR (Thermo Nicolet model Nicolet 6700) was performed using the KBr disc method. Raman spectra were collected using a UHTS 300 Raman spectrometer (WITec, Germany) with an excitation wavelength at 532 nm. The structures of the GO nanocarrier and GOGA nanocomposite were observed on a high-resolution transmission electron microscope (HRTEM), Tecnai G2 (FEI, USA). The samples were prepared by placing a drop of a sonicated dispersion on a carbon grid and dried at 37 °C for 24 h. The controlled release properties and the loading capacity were studied using a Lambda 35 spectrophotometer (PerkinElmer, Boston, MA).

### Preparation of GO Nanocarrier and GOGA Nanocomposite

Graphene oxide was synthesized from graphite (Gr) by the improved Hummers method [30]. A 9:1 mixture of concentrated H_2_SO_4_/H_3_PO_4_ (360:40 mL) was added to a mixture of graphite flakes (3.0 g, 1 wt equiv) and KMnO_4_ (18.0 g, 6 wt equiv). The reaction was then heated to 50 °C and stirred for 12 h. The reaction was cooled to room temperature and poured onto ice (400 mL) with 30 % H_2_O_2_ (3 mL). The obtained suspension was centrifuged at 4000 rpm for 5 min and then washed with 200 mL of deionized water (DIW), 200 mL of HCl (37 %), and then with 200 mL of ethanol, respectively. The obtained product was coagulated with 200 mL of diethyl ether and then filtered by Omnipore™ membrane with a 0.2-μm pore size. The obtained GO on the filter was dried in an oven at 40 °C [30]. A 1 % of pure drug (GA) was dissolved in DIW and 0.1 g of GO was added and the pH fixed at 4.71. The mixture was stirred for 16 h and then washed with DIW to remove unreacted drug. The GOGA nanocomposite was collected via filtration and dried in an oven at 40 °C.

### In Vitro Cytotoxicity Experiment

The normal mouse fibroblast cell lines 3T3 and human hepatocellular liver carcinoma cell lines HepG2 were obtained from American Type Culture Collection (ATCC, VA, USA), and these cells were cultured in DMEM and RPMI medium (Nacalai, Japan) supplemented with 10 % (*v*/*v*) fetal bovine serum (FBS) and 1 % antibiotic solution, respectively. To determine the cell viability, healthy viable cells were seeded to cell culture plates (1 × 10^4^) once reach the 80 % of confluency in a cell culture flask and seeded cells were allowed to adhere overnight earlier to the nanocompound treatment with different doses (0.781–50 μg/mL). The cells without any treatment were served as a negative control, and another set of cells were treated with vehicle control (0.1 % dimethyl sulfoxide (DMSO)). Treated cells were incubated for 72 h; after the incubation period, cell counting reagent (CCK-8 solution; Dojindo Lab, Japan) was then added to each cell culture well and placed in an incubator for 3 h at 37 °C in a humidified atmosphere with 5 % CO_2_. After the incubation period with cell counting reagent, the 96-well cell culture plates were measured at 450 nm by a microplate reader (BioTek Instruments Inc., VT, USA). The cell viability percentage was determined according to the kit protocol, and cell viability percentage was plotted on a graph.

### Drug-Release Procedure

To study the controlled-release characteristics of GA loaded on GOGA nanocomposite, a pH 7.4 solution was used, which has a similar pH of blood. Various studies show that different anions such as HPO_4_^−2^ and H_2_PO_4_^−^ have an affect on the rate of drug release [31–33]. The release rate of GA from GOGA nanocomposite was carried out by adding 14 mg of GOGA nanocomposite into 10 mL of phosphate-buffered solution (pH = 7.4) and used after 10 days. The accumulated release amount of GA from GOGA nanocomposite was measured at *λ*_max_ = 264 nm at room temperature.

## Results and Discussion

### X-ray Diffraction

**Fig. 1:**
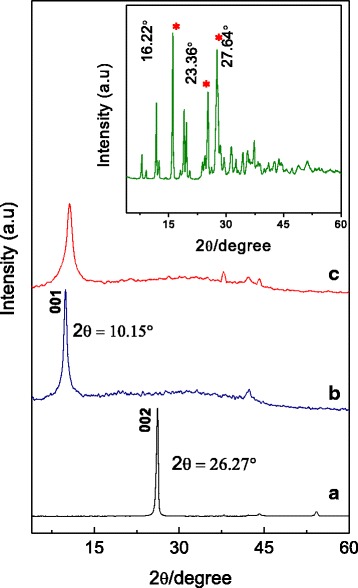
XRD patterns of Gr (**a**), GO nanocarrier (**b**), and GOGA nanocomposite (**c**). The *inset* shows the XRD patterns of pure GA

Figure 1 depicts XRD patterns of pristine graphite (Gr), GO nanocarrier, and GOGA nanocomposite. The diffraction pattern of pure Gr (Fig. 1(a)) shows a very intense, sharp peak at 2*θ* = 26.27°, attributed to the diffraction of the (002) graphite plane composed of highly organized layers with an interlayer spacing of 0.34 nm [34, 35]. After oxidative exfoliation of Gr, the characteristic peak of Gr was no longer visible and a new strong sharp peak at 2*θ* = 10.15° was observed which is attributed to the diffraction of the (001) for GO (Fig. 1(b)). An increase in the interlayer distance of GO (0.87 nm) might be due to the exfoliation of Gr layers and the formation of oxygen-containing functional groups such as hydroxyl, epoxy, and carboxyl [36], as well as the intercalated water molecules on the surface of GO interlayer’s [37, 38]. The inset shows the XRD patterns of pure GA with several intense and sharp crystalline peaks at 2*θ* values of 16.22°, 25.36°, and 27.64°, corresponding to the characteristic of an organic molecule with crystalline property [39]. In contrast, GOGA nanocomposite (Fig. 1(c)) shows amorphous characteristic, lacking of crystalline peaks in contrast to the XRD patterns of pure drug, GA. Since the characteristic peaks of GA were not observed in the XRD patterns of GOGA nanocomposite, it proved that the loading of the drug to the GO carrier has taken place. The successful formation of GOGA nanocomposite was further confirmed by other complimentary techniques such as FTIR, Raman, and UV-visible spectroscopy, which will be discussed in other sections. Moreover, the result shows that there are no big difference between the diffractograms of GO and GOGA nanocomposite, confirming that the loading procedure did not affect the phase change in the resulting GO nanocarrier.

### Infrared Spectroscopy

**Fig. 2:**
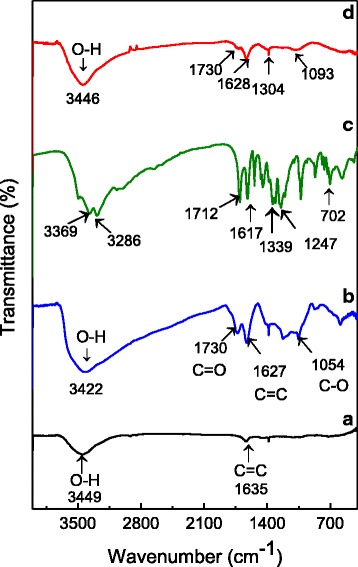
Fourier transform infrared spectra for **a** Gr, **b** GO, **c** GA, and **d** GOGA nanocomposite

Figure 2 shows the FTIR spectra of pure Gr, GO, and GOGA nanocomposite. Pure Gr spectrum (Fig. 2(a)) shows a broad peak at 3449 cm^−1^ attributed to the existence of moisture in the pristine Gr [34]. The peak at 1635 cm^−1^ is assigned to stretching vibrations of C=C bonds. Figure 2(b) shows an intense broad peak for GO nanocarrier at 3422 cm^−1^ due to the stretching –OH band. The existence of –OH groups in GO can be bonded to the various sites of the carbon skeleton, resulting in the broadening of the peak [40]. The peak at 1730 cm^−1^ is attributed to the stretching vibration of C=O bonds presence in carboxylic acid and carbonyl groups. The low intensity C=C bonds at 1627 cm^−1^ corresponding to remaining sp^2^ character of graphite [41]. In addition, the peaks in the region of 1362 to 1049 cm^−1^ might be due to the COC/COH bonds [40]. Figure 2(c) indicates the characteristic bands of pure GA at 3286 cm^−1^ (acidic O–H stretching), 1712 cm^−1^ (presence of phenol group), 1617 cm^−1^ (C=C stretching vibration of aromatic ring), 1247 cm^−1^ (presence of carboxylic groups), 1026 cm^−1^ (C–O stretching of carboxylic group), and 731 cm^−1^ (δ_CC_ benzene ring vibrations) [42, 43].

The peak observed in Fig. 2(d) for GOGA nanocomposite at around 3446 cm^−1^ can be assigned to the O–H functional groups from carboxyl or phenols. After loading of the drug, GA into GO nanocarrier with the characteristic bands of 1730 and 1628 cm^−1^ with lower intensity still remained. The infrared spectrum of GOGA nanocomposite (Fig. 2(d)) shows the characteristic peaks of both GO nanocarrier and GA which suggest the successful formation of the nanocomposite.

### Raman Spectroscopy

**Fig. 3:**
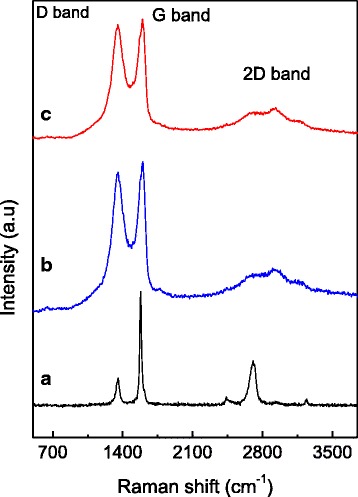
Raman spectra of Gr (**a**), GO (**b**), and GOGA nanocomposite (**c**)

Raman spectroscopy was used to analyze the disorder and defects in crystal structure of graphite and its derivative (GO). In order to obtain disorder, the intensity ratio between the disorder-induced D band and the G band (I_D_/I_G_) can be measured. Previous works proved that the I_D_/I_G_ ratio strongly depends on the amount of disorder in the graphitic material [44]. The Raman spectrum of Gr, GO, and GOGA nanocomposite is shown in Fig. 3. It is clear that significant structural changes took place during the chemical transformation from Gr to GO (Fig. 3(a, b)). Usually, all sp^2^ carbon forms have the G band, which appears from the first-order Raman scattering [45–47]. The G band is observed at 1579, 1601, and 1599 cm^−1^, while the D band is distinguished at 1352, 1355, and 1353 cm^−1^ for Gr, GO, and GOGA nanocomposite, respectively. The D band corresponded to the presence of disorder in the sp^2^ carbon network, and broadening of D bands in GO can be due to a decrease of the sp^2^ domain size, caused by the creation of defects, vacancies, and distortions during oxidation [48]. The 2D band (G′ band) appears at 2706, 2902, and 2933 cm^−1^ in Raman spectrum corresponding to the second-order dispersive Raman mode for the Gr, GO, and GOGA nanocomposite, respectively [46, 49]. Raman spectra of GO and GOGA nanocomposite (Fig. 3(b, c)) show no significant shift in the D band and G band. The increase of I_D_/I_G_ of the GO nanocarrier (0.96) when compared to the Gr (0.84) confirms that grafting of oxygen-containing functional groups to the graphitic planes has taken place [48].

### Morphology Study Using HRTEM

**Fig. 4:**
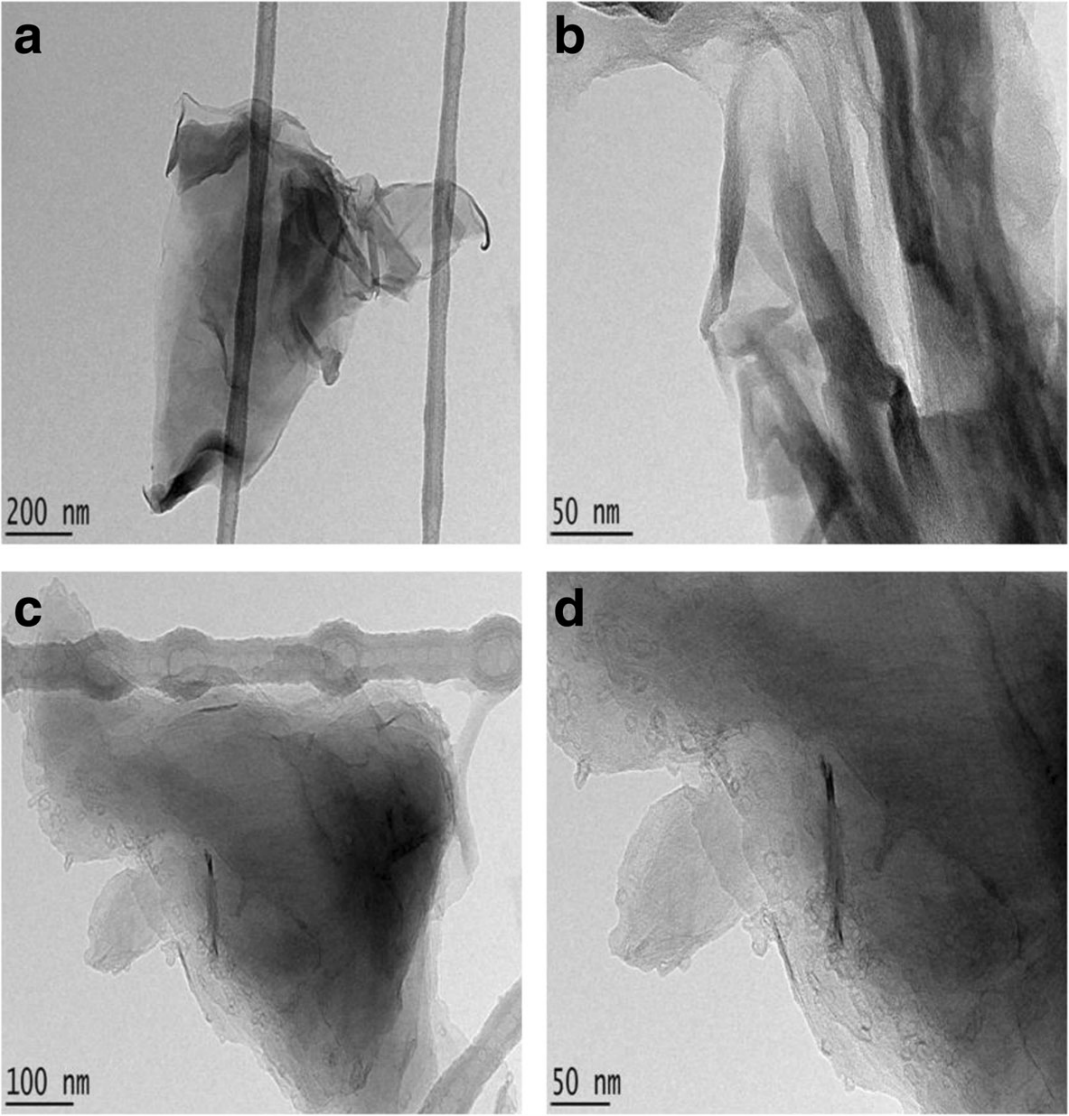
HRTEM images of **a** GO nanocarrier with 200 nm bar, **b** GO nanocarrier with 50 nm bar, **c** GOGA nanocomposite with 100 nm bar, and **d** GOGA nanocomposite with 50 nm bar

High-resolution electron microscopy (HRTEM) was used to further elucidate the morphology and dispersion of the compounds. Figure 4 shows the HRTEM images of the GO nanocarrier and GOGA nanocomposite. It was revealed that the large sheets in GO (Fig. 4a, b,) tend to agglomerate to each other, forming a multilayered agglomerate. These sheets resemble a wavy silk veil, transparent, and entangled with one another [40]. The HRTEM images of GOGA nanocomposite (Fig. 4c, d) show GO forms similar to agglomerate multilayer sheets, with the drug loaded into the surface of GO nanocarrier.

### Loading and Release Behavior of GA

**Fig. 5:**
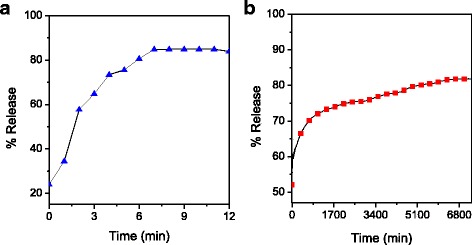
Release profiles for **a** physical mixture of GA and **b** GOGA nanocomposite in phosphate-buffered solution at pH 7.4

The release profiles of GA from a physical mixture of (GO-GA) and the GA from GOGA nanocomposite into phosphate-buffered solution at pH 7.4 (blood pH) are shown in Fig. 5. The fast release of GA from the physical mixture was completed within 7 min at pH 7.4 (Fig. 5a). This is due to the low electrostatic attraction between the GA anions and GO nanocarrier. Figure 5b shows that the cumulative release rate of GA into buffered solution at pH 7.4 is significantly slower and sustained which is presumably due to the anion-exchange process taking place between the GA anions and the anions in the buffered solutions [50]. In addition, the initial burst release [51] observed in the first 1 h (59 %) can be due to the surface characteristics of GO material, the drug interactions, or/and morphology and porous structure of the material [51]. The maximum percentage release of GA from the GOGA nanocomposite reached 81 % in about 7200 min (or 5 days) at pH 7.4. From the calibration curve equation and using the ultraviolet instrument, 12 % GA loaded to the GO nanocarrier can be obtained. As such, the percentage of GA released from GOGA nanocomposite at equilibrium did not reach 100 %, which can be attributed to the characteristics of the ion-exchange reaction mechanism, i.e., the loaded anions cannot be exchanged completely at equilibrium, but the organic species released was removed continuously [52]. These results show that the GOGA nanocomposite has a good potential to be used as a drug delivery system with controlled sustained release property.

### Release Kinetics of GA from GOGA Nanocomposite

The kinetic release of GA from the GOGA nanocomposite was fitted using a number of different kinetic models, such as pseudo-first order (Eq. 1), pseudo-second order (Eq. 2), parabolic diffusion (Eq. 3), Higuchi Model (Eq. 4), and Korsmeyer-Peppas Model (Eq. 5) [53, 54]. The equations are shown below

1 $$ \ln \left({q}_e - {q}_t\right) = \ln\ {q}_e - kt $$

2 $$ t/{q}_t=1/k{q_e}^2+t/{q}_e $$

3 $$ \left(1 - {M}_t/{M}_0\right)/t = k{t}^{-0.5} + b $$

4 $$ {q}_t=K\sqrt{t} $$

5 $$ {q}_t/{q}_{\infty } = K{t}^n $$

where *k* is the corresponding release amount constant, *q*_*e*_ and *q*_*t*_ are the equilibrium release amount and the release amount at time *t*, respectively, and *M*_0_ and *M*_*t*_ represent the drug (GA) content remaining in the GO nanocarrier at release times 0 and *t*, respectively.

**Fig. 6:**
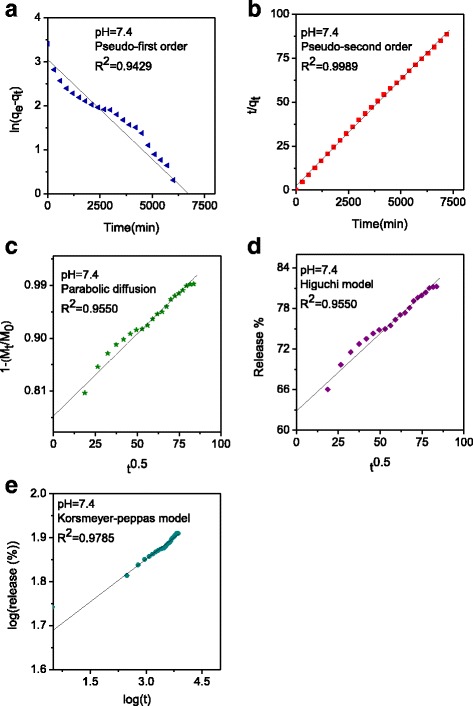
Fitting the data of GA release from GOGA nanocomposite at pH 7.4 for kinetic models **a** pseudo-first order, **b** pseudo-second order, **c** parabolic diffusion, **d** Higuchi model, and **e** korsmeyer-peppas model

The Higuchi equation plot (Fig. 6d) with correlation coefficient (*R* ^2^ = 0.9550) shows the release of the drug from the GOGA matrix a square root of time and is dependent on Fickian diffusion. The results (Fig. 6 and Table 1) indicated that the release of the active drug, GA, from the GOGA nanocomposite followed the pseudo-second-order kinetic model, with a best fit value for the correlation coefficient (*R* ^2^ = 0.9989), as shown in Fig. 6 and Table 1. In addition, the *t*_1/2_ time release of GA into phosphate-buffered solution at pH 7.4 was 203 min using pseudo-second-order kinetic model (Table 1).

**Table 1:** Correlation coefficients (*R* ^2^), rate constant (*k*), and half-time (*t*_1/2_) obtained by fitting the GA release data from the GOGA nanocomposite into phosphate-buffered solution at pH 7.4

Aqueous solution	Saturated release (%)	*R* ^2^					Rate constant (*k*)^a^ (mg/min)	*t*_1/2_^a^ (min)
		Pseudo-first order	Pseudo-second order	Parabolic diffusion	Higuchi model	Korsmeyer-peppas model		
pH 7.4	83.1	0.3294	0.9832	0.9550	0.9550	0.9785	2.42 × 10^−4^	203

^a^Estimated using pseudo-second-order kinetics

### Inhibition of Cancer Cell Growth by the Nanocompound

**Fig. 7:**
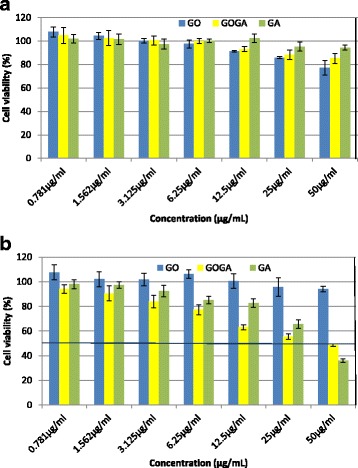
Cytotoxic effects of GO, GOGA, and GA on the normal fibroblast cells and HepG2 liver cancer cells. Normal fibroblast cells 3T3 (**a**) and HepG2 (**b**) cells were treated with different doses of carrier, pure drug, and GOGA nanocomposite for 72 h and cytotoxicity was measured using a CCK-8 assay kit and the results were calculated according to the kit protocol and results are expressed as mean ± SD

The potential cytotoxicity of GO carrier, GA, and GOGA nanocomposite on proliferation of normal fibroblast cells and cancer cells on preliminary screening results is shown in Fig. 7. In the normal fibroblast cells (Fig. 7a), we did not notice any cytotoxicity effect up to the highest concentration of 50 μg/mL. Therefore, the GOGA nanocomposite is safe to use for further experiments. In the liver cancer cells (Fig. 7b), the percentages of viable cells were decreased in a dose-dependent manner. The results show that GO nanocarrier had a negligible effect in liver cancer cells, with almost 100 % of cells remaining viable at 50 μg/mL compared with about 48 % of cells remaining viable for GOGA nanocomposite. Thus, the cytotoxicity to the liver cells is likely attributable to the release of GA from the GO carrier rather than the effect of the carrier itself. This result shows that the anticancer activity of our new nanocomposite is very similar to that of pure GA and suggests the possibility of a decreased dosing interval due to the sustained-release ability of the nanocomposite. With the sustained release and possible targeted delivery potential of GOGA nanocomposite, the least amount of active agent (GA) could suffice, hence reducing the dosing interval and unnecessary exposure to large quantities of this hazardous drug.

The results further suggest the possibility of increased anticancer activity with increasing the loading percentage. The IC_50_ values are 429.5, 42.9, and 38.9 μg/mL for GO nanocarrier, GOGA nanocomposite, and GA, respectively. These results have suggested that GO-based nanocarrier is one of the good candidates for drug delivery system against cancer cells, without showing noticeable cytotoxicity in normal fibroblast cells.

## Conclusions

In this study, we report the design and synthesis of an anticancer nanodelivery systems based on a graphene oxide-gallic acid nanocomposite. The empty carrier GO and designed anticancer nanocomposite (GOGA) was found to be highly biocompatible with normal fibroblast cells. The designed GOGA nanocomposite showed good anticancer activity against liver cancer (HepG2) cells. The results show that cytotoxicity to the liver cells is likely attributable to the release of GA from the carrier rather than the effect of the carrier itself. And due to the similar anticancer activity of our new nanocomposite to that of pure GA, the possibility of a decreased dosing interval due to the sustained-release ability of the nanocomposite can be suggested. Therefore, the least amount of active drug (GA) could suffice, hence reducing the dosing interval and unnecessary exposure to large quantities of this drug. Sustained release of active agent (GA) over longer period of time (6800 min) will increase the viability of drug and would ultimately result in better therapeutic efficacy and decreasing in drug dosing frequency. These in vitro studies have encouraged this research towards the next level of in vivo studies.

## Abbreviations

DMSO: Dimethyl sulfoxide
FTIR: Fourier transform infrared
GA: Gallic acid
GO: Graphene oxide
GOGA: Graphene oxide which loaded with gallic acid
GO-GA: Physical mixture of graphene oxide-gallic acid
Gr: Graphite
HRTEM: High-resolution transmission electron microscopy
PBS: Phosphate-buffered saline
XRD: X-ray diffraction
